# Chinese health literacy scale for tuberculosis patients: a study on development and psychometric testing

**DOI:** 10.1186/s12879-019-4168-z

**Published:** 2019-06-20

**Authors:** Yan Li, Shaoru Zhang, Tianhua Zhang, Yi Cao, Weiping Liu, Hualin Jiang, Dan Ren, Jing Ren, Haini Liu, Zhongqiu Hua

**Affiliations:** 1 Department of Nursing, Xi’an Jiaotong University, Xian, 710061 China; 2 School of Nursing, Lanzhou University, Lanzhou, 730000 China; 3 Shaanxi Provincial Institute for TB Control and Prevention, Xian, 710048 China

**Keywords:** Tuberculosis patients, Health literacy, Scale, Psychometric testing

## Abstract

**Background:**

The role of health literacy on tuberculosis patients has not been evaluated in China, in part because few special health literacy measurements exist.

**Methods:**

A three-step design process was used: (1) Scale construction: Based on the model of revised Bloom’s taxonomy, the item-pool was drafted from a literature review, focus group discussion, and in-depth interviews. In addition, a Delphi survey was used in order to select items for inclusion in the scales; (2) Pilot study: Acceptability and clarity were tested with 60 tuberculosis patients; and (3) Psychometric testing: Validity analysis includes content validity, construct validity, and discriminative validity. The Cronbach’s alpha coefficient, split-half reliability, and test-retest method were used to assess reliability. Finally, a receiver operating characteristic analysis was conducted to generate a cut-off point.

**Results:**

The final scale had 29 items with four domains. The item level Content Validity Index ranged from 0.70 to 1.0, and the scale level Content Validity Index was 0.95. The mean score among the lowest 27% group was significantly lower than that those of the highest 27% group (*p* < 0.01), which supports adequate discriminant validity. Explanatory factor analysis produced a clear four-factor construct, explaining 47.254% of the total variance. Factor 1 and Factor 2 were consistent with read and memorize TB-related words; Factor 3 was associated with understand the meaning of the health education leaflets and examine if TB patients can apply the correct approach to correct context; Factor 4 was related to the ability of TB patient to calculate and identify what unspecified assumptions are included in known conditions. The confirmatory factory analysis results confirmed that a four-factor model was an acceptable fit to the data, with a goodness-of-fit index = 0.930, adjusted goodness of fit index = 0.970, root mean square error of approximation = 0.069, and χ2/df = 2.153. The scale had good internal consistency and test-retest reliability. Additionally, the receiver operating characteristic analysis indicated that the cut-off point for the instrument was set at 45 and 35.

**Conclusions:**

The Chinese Health Literacy scale for Tuberculosis has good reliability and validity, and it could be used for measuring the health literacy of Chinese patients with tuberculosis.

## Background

### What is health literacy?

Health Literacy (HL), as a new term, was first proposed by American scholar Simonds [1]. Since then, the term HL began to receive attention from countries around the world and was gradually introduced into various researches. Many scholars and institutions, including World Health Organization (WHO), the Institute of Medicine (IOM), the European Union (EU), etc., have explained and defined it. In 1998, the WHO defined HL as follows: “HL reflects a person’s social and cognitive skills, which in some ways determine whether an individual has the ability to understand, apply, analyze, and disseminate health information or health services, and whether an individual can maintain and promote health through these skills” [2]. In 2004, the IOM defined HL as follows: “HL is not only the ability to read health materials, but also a skill to obtain, understand, adopt and implement relevant health information from various platforms” [3]. In 2007, the EU defined HL as “the ability to read, filter and understand health information to form sound judgment” [4]. In conclusion, the definition of health literacy has been constantly evolving. HL includes not only basic skills such as reading, writing, computing, and communication when individuals interact with a healthy environment, but also the ability to apply these skills to solve health problems, make appropriate health decisions, reduce health-related risk factors, so that the individual is always in the best state of health. Therefore, people with adequate HL should: (1) be able to understand and practice health promotion and disease prevention; (2) have the necessary health awareness and basic health knowledge; (3) master the necessary health skills; (4) can understand their own conditions and treatment methods, make appropriate decisions for their health; (5) can take the right medicine at the right time according to the predetermined dose; (6) can master reading, writing, calculation and basic communication skills, in order to obtain, evaluate and practice health information; (7) can advocate the health of individuals, families and communities [5–7].

### The role of health literacy on tuberculosis and critical reasons to measure it

China is ranked 3 among the top 22 countries with high tuberculosis (TB) burden, accounting for 10% of the total global TB incidences [8]. In the 1990s, China implemented a TB control program based on directly observed treatment, short-course (DOTS) strategy [9]. The prevalence of smear-positive TB fell by 65% from 1990 to 2010 [10]. These gains enabled China to achieve the 2015 global TB control target of reducing the prevalence of smear-positive TB by 50%. In fact, China is the only country with a high rate of TB burden to achieve such goal [10]. However, global health conditions, TB care and TB prevention have entered a new era. In May 2014, the 67th World Health Assembly adopted the World Health Organization’s ‘Global strategy and targets for TB prevention, care and control after2015’ (End TB Strategy), with a goal of a 90% reduction in incidence and 95% reduction in mortality by 2035 [11, 12]. Thus, more effective solutions are urgently needed to further reduce TB burden in China.

The most important feature of TB is that it can be prevented, controlled, and cured. Successfully managing TB infection requires each individual patient to access information of medical care, to understand reason for recommendation, to execute treatment plans, to remember drug labels, and to interpret correctly the test results, doses of drugs, and informed consent documents. These skills and knowledge may be influenced by the individual’s health literacy. Evidence from the U.S. has shown that low health literacy is costly and causing poor health outcomes [13]. On the patient side, there is additional expenditure per year per person with limited health literacy compared to that in persons with adequate health literacy [14]. A report from the IOM institute indicated that tens of millions of U.S. adults were unable to read complex text, including many health-related materials [3]. Groups with higher rates of low health literacy included patients from remote areas, patients having lower income or low education, patients with age over 65, were generally in poor health conditions [15, 16]. Compared to those with adequate health literacy, individuals with limited health literacy had less knowledge about disease and treatment plans [17], less ability to correctly manage medication [18–20], more problems in communicating with health care professionals [21, 22], poorer self-care skills [23, 24]. Patients with limited health literacy have high risk of delaying their decision to seek medical care, and experiencing negative health outcomes [25–30].

With the increasing focus upon patient-centered approaches and augmentation of patients’ self-care skills, the concept of health literacy has become highly relevant in medical care settings. At the clinical side, health care professionals need to know the capacity of patients to process and understand health information so that effective communication may occur [31]. At a community setting, in order to develop appropriate policies and provide sufficient resources, policy-makers need to understand the patient’s ability to receive and understand health information [31]. Measurement is a fundamental activity of science [32]. We acquire knowledge about people, objects, events, and processes by observing them, and making sense of these observations often requires us to quantify them [32]. The process of measurement and the broader scientific questions it serves interact with each other [32]. A disease-specific or context-specific health literacy measurement may be more effective when applied to populations in need of managing a particular illness or condition [3]. Furthermore, health literacy data can help health professionals to evaluate health education needs, as an effective evaluation tool to guild appropriate disease-specific intervention. There are a number of related studies on health literacy measurements for specific diseases such as type 2 diabetes [33–35], hypertension [36–38], asthma [39, 40], and AIDS [41–43]. However, similar study on TB is not yet available.

### Available measurements of health literacy

A recent summary of health literacy measurements evaluated 51 studies and concluded that none of them appeared to measure a comprehensive set of skills that were conceptualized as the necessary components of health literacy [44]. Additionally, the content of these measurements of health literacy skill levels has focused on the ability to read and, in some cases, to use numbers [45]. The instruments most commonly used to measure health literacy include Rapid Estimate of Adult Literacy in Medicine (REALM) [46], Test of Functional Health Literacy in Adults (TOFHLA) [47], and National Assessment of Adult Literacy (NAAL).

The REALM was developed in the United States to assist physicians to identify adults with limited reading skills in the primary care settings. Word recognition test assesses whether a person can correctly pronounce a series of health-related words listed sequentially with increasing difficulties. The REALM emphasizes language capability, which reflects people’s ability to recognize terms, but ignoring their understanding and skills in health literacy. REALM is currently one of the most commonly used health literacy measurements. It was developed in 1991 by American scholar Davis and others. As the world’s first tool for measuring health literacy, REALM is suitable for adult over the age of 18. REALM identifies patients with limited reading ability by assessing their understanding of medical-relevant terms, and then rearranges health education materials or colloquial health education according to their ability. Another commonly used health literacy measurement tool is TOFHLA, developed by Parker et al. in 1995. This scale is mainly used in the medical background. It consists of 17 arithmetic questions that evaluate the patient’s computational ability and 50 cloze questions that evaluate the patient’s reading ability. It takes an average of 22 min for each patient to complete this scale, and the investigator needs to train for a long time. In addition to these common measures, another relatively new tool for health literacy assessment is the NAAL developed by the United States in 2004. The tool is a polynomial answer, and the respondent needs to answer by thinking and judging. It mainly tests the short-term literacy, document literacy, and arithmetic literacy that the respondent has in the relevant health field. It did not take into account the ability to communicate with doctors, or understanding of medical terms. China carried out a National Health Literacy Survey in 2007 to understand the level of basic health knowledge and skills in Chinese population [48]. A composite index and percentages were adopted to assess health literacy. The project mainly assessed knowledge levels and health-related behaviors of Chinese residents, but did not sufficiently reflect their health literacy skills.

All these studies mentioned above are not designed specifically for patients with TB, who usually need to remembering TB-related words, understand the relevant concepts such as cause, symptoms, and signs of TB, remember treatment and management of TB infection with skills to read medication labels, doses and warnings, to interpret the test results, to use educational brochures, and to apply these in health decision-making.

### Aims of the study

As discussed above, health literacy may have a notable impact on managing TB infection. Such profound levels of limited health literacy may impact the ability of individual to self-manage diseases like TB infection. Impact of limited health literacy on health outcomes needs to be evaluated too. There is no special measurement to evaluate health literacy on TB. As such, the aim of this study was to develop a Chinese Health Literacy Scale for Tuberculosis (CHLS-TB), and to investigate its psychometric properties.

## Methods

### Scale development

#### Phase 1: Theoretical framework

The CHLS-TB was developed with reference to the revised Bloom’s taxonomy model [49]. Bloom’s taxonomy model was recently adopted as the framework in the research team’s development of the other two health literacy scales. One measured the ability of Chinese patients with Type 2 diabetes mellitus to recognize diabetes-related concepts and apply these concepts in health decision-making [50]. The other was developed for chronic cares [51]. The present study continued this effort to combine the revised Bloom’s taxonomy model and health literacy, playing a guiding role in the establishment of a scientific system of health literacy theory and evaluation tools.

#### Phase 2: development of an item pool

1. Literature review. A comprehensive literature search through PubMed, Web of Science and Google Scholar to identify concepts of TB and health literacy and its components, as well as related scales using the Keywords: “TB patients skill”, “TB patients literacy”, “health literacy”, “TB patients health literacy”, “health literacy scale”, organizing health literacy evaluation contents for TB patients.
2. Material collection. The research team stayed 2 weeks at TB specialist clinics in Shaanxi Province, China; all TB patients received medical consultation and follow-up care in these clinics. During the process, the research team identified some important words, skills, common practices and issues in the context of TB care, and gathered commonly used drug labels, health education leaflets and information sheets to TB patients.
3. Focus group discussion (FGD). We ran 3 focus group discussions to gain a comprehensive understanding of the health literacy needs in the context of TB care in the target populations. These included 2 FGD for medical staff (6 persons), 1 FGD for family members of TB patients(6 persons). The discussed topics were (a) basic TB-related treatment words that needed to read and remember; (b) the correct number and time of drug pills to take daily; and (c) difficulties encountered in interpreting the test results, educational brochures or drug labels, etc.
4. In-depth interviews. Under the principle of informed consent, confidentiality and voluntariness, the research team adopted the convenient sampling method, recruited 20 TB patients (10 outpatient TB patients, 10 hospitalized TB patients) for the personal in-depth interview. The content of interview included: (a) the patient’s current physical condition; (b) the source of health information; (c) problems encountered in communicating with health care providers; (d) the knowledge and familiarity of patients with drug labels, health education leaflets and examination results; and (e) the patient’s medical behavior, the situation of taking the medicine and problems encountered during treatment.

#### Phase 3: item selection

In this study, Delphi method was used to screen the items included in the CHLS-TB. The item-pool of scale, was evaluated by 10 experts with expertise in TB control and care in China. In each round, experts were asked to rate each item according to the five-point Likert scale, with a score range of (most relevant to CHLS-TB) to 1 (least relevant). In addition, they are asked to evaluate the accuracy, relevance, and readability of each item by choosing Yes or No. The content validity index (CVI) of was calculated for each item. The CVI for an item is the proportion of experts who rate it as 4 or 5. A CVI value of >0.80 was set the cut-point for acceptable validity [52]. Informed consent was obtained at the start of the Delphi round.

### Pilot study

A pilot study of the CHLS-TB was conducted in Xi’an Chest Hospital, Shannxi Province, China. A sample of 60 TB patients was recruited using a convenient sampling method and their written informed consent was obtained. The purpose of this pilot study is: a) to understand whether the items in the scale are clear and ambiguous; b) modify or delete unnecessary words to reduce the burden on the patient’s reading; c) to evaluate whether the wording of the scale is suitable the actual language of the patient’s daily situation; d) to understand the patient’s acceptance of the scale and the time required to complete the scale.

### Assessing the psychometric properties of the scale

#### Study design

We used a cross-sectional study design to test the psychometric properties of the CHLS-TB.

#### Participants

A convenience sampling strategy was used to recruit participants having tuberculosis from the six locations of Shannxi Province, China (Xi’an Chest Hospital, Shaanxi Province Tuberculosis Hospital, two community and two primary healthcare settings), between December 2016 and June 2017. The sample inclusion criteria are: a) ≥18 years old, clinically diagnosed as a TB patients; b) voluntarily participate in the study and sign the informed consent; c) have normal communication skills. The sample exclusion criteria are: a) those who do not meet the above inclusion criteria; b) those with cognitive impairment or mental disorders;

The size of the sample in this study was determined mainly based on the number of items in the developed scale and the sample size requirements of factor analysis. The sample size should be 5 to 10 times the number of items in the scale. In this study, 30 design items are proposed, so the sample size is at least 150. Considering the invalid questionnaire, increase the sample size by 20%, and the sample size is *n* ≥ 180. Samples for exploratory factor analysis (EFA) and confirmatory factor analysis (CFA) should be two independent samples, preferably with the sample size larger than 100 and 200 respectively [53, 54], so the minimum sample size required for factor analysis is 300. Considering the invalid questionnaire, increase the sample size by 20%, and the sample size is *n* ≥ 360. In consideration of the above, the sample size of this study should be no less than 360. According to the inclusion and exclusion criteria, a sample size of 521 participants was recruited.

#### Ethics statement

The research was approved by the Institutional Review Board of Xi’an Jiaotong University (Letter Number: 2017–518). Written informed consent was obtained for all research participants, and informed that they could withdraw from the study at any time. All questionnaires were completed anonymously, and the information obtained was only used for this study and kept strictly confidential.

#### Data collection

The members of the research group were divided into two groups, and two members went to the target investigation site as a team. With the assistance of the medical departments of all hospitals, community, and primary healthcare settings, the questionnaires were distributed to inpatients and outpatients by investigators. The issuance and filling of the questionnaires were conducted by the investigators on a one-to-one basis, and the investigator is required not to take any subjective emotion and not to use any suggestive and inducing language.

#### Statistical analysis

The database was built using Epidata3.0 software, and the data was analyzed using SPSS 22.0 and Amos18.0 software. Continuous variables were presented in mean and standard deviation (SD). Categorical variables were presented as numbers and frequency. Psychometric properties of the CHLS-TB were reflected by validity and reliability. We also present receiver operating characteristic (ROC) analyses that provide estimates of various scores’ sensitivity and specificity so that the CHLS-TB can be used as a screening measure. Generally, the α level was set at 0.05.

1. Content validity

The content validity index (CVI) calculated in the final round of Delphi survey was used to evaluate the content validity of the scale.

(2) Construct validity

Exploratory and confirmatory factor analysis (EFA and CFA) were used to examine the construct validity of the scale, with the total samples splitting into two sub-samples of near-equal size through random selection of cases (using the automated function in SPSS 22.0). One group participants (N1 = 253) was used to conduct the EFA, and the other group (N2 = 245) was used for CFA. The samples were examined for equivalence on demographic characteristics using the Mann-Whitney U tests (for continuous variables) and Chi-Square tests (for categorical data).

1. Exploratory factor analysis

Before performing EFA, the sample data used should be tested. If the Kaiser-Meyer-Olkin (KMO) value is <0.6 [55] and the Bartlett spherical test result is not significant (P>0.05) [56], it means that the sample data is not suitable for factor analysis. In this study, the principal component factor analysis was used to determine the common factors. The number of common factors was determined according to the following criteria: (a) eigenvalues > 1.0, (b) scree plot [57]. Then, the maximum orthogonal rotation of the variance is performed on the obtained factor load matrix, so that the common factors have clear professional significance, and the factor structure model of the scale is further clarified.

(2) Confirmatory factor analysis

CFA was performed to test whether data fit the hypothesized measurement model, which was extracted by EFA. Maximum likelihood estimation method was used at CFA. Model fitness was assessed by the goodness-of-fit index (GFI), adjusted goodness of fit index (AGFI), root mean square error of approximation(RMSEA), and the normed chi-square (χ2/df). For GFI and AGFI, a value closer to 1 indicates better fit, for RMSEA, a value of < 0.10 is considered to be acceptable, and for χ2/df, a value of *<* 3 is suggested as the criteria for optimal fit [58].

(3) Discriminant validity

Discriminant validity refers to a scales ability to distinguish between two or more groups that it should be able to distinguish between. By using this scoring method, every respondent earned a total score after completing the questionnaire. Then two independent samples t-test were conducted to compare the mean score of the highest 27% (P_H_) to the lowest 27% (P_L_) of the respondents to test the discriminative efficiency of scale in assessing the TB patients’ health literacy. If *p* < 0.05, the scale was considered discriminatively efficient.

(4) Reliability

Reliability of the scale was assessed using internal consistency reliability and test-retest reliability, and it was conducted on the full sample. The internal consistency reliability was judged by Cronbach’s alpha coefficient and split-half reliability. It was completely unacceptable when the Cronbach’s alpha coefficient is lower than 0.60. The split-half reliability was performed after all the TB patients complete the scale, the items of the scale were divided into two halves according to the odd and even numbers of the items number, and the correlation coefficients of the two half scores are calculated. In order to evaluate the test-retest reliability, 50 TB patients were randomly selected from the total participants in this study, and the measurements were taken again 2 weeks later, and then the Pearson relation analysis were conducted. The test-retest reliability was acceptable when the correlation coefficient was higher than 0.75 [59].

(5) Receiver operating characteristic (ROC) analysis

The primary aim of CHLS-TB was to identify if someone has low levels of health literacy, therefore a clinically meaningful cut-off point of the CHLS-TB score that separates low health literacy from adequate health literacy is pivotal. This was done through ROC curve analysis based on the TB patients’ educational level. The ROC curve analysis was also conducted on the full sample.

## Results

### Scale development

#### Phase 1: Theoretical framework

According to the revised Bloom’s taxonomy model, we divided the whole goal of health literacy in TB patients into four processes: remembering, understanding, applying, and analyzing, respectively corresponding to the four dimensions of the scale. The “remembering” is mainly about reading and memory, requiring TB patients to recognize TB-related words and store it in the brain; the “understanding” requires that the TB patient be able to master the meaning of the health education leaflets, drug labels or checklist, and when asked about the use of these concepts, they can be correctly explained; the “applying” is to examine TB patients in a new context, without suggesting what kind of practice is correct, he can apply the correct approach to the correct context; the “analysing” evaluates the ability of the TB patient to calculate and identify what unspecified assumptions are included in known conditions.

#### Phase 2: development of an item pool

Using these combined methods at the second phase of the study, we developed a comprehensive pool of 133 items most commonly used in the context of TB care. A total of 101 terms were chosen to measures an individual’s ability to read and memorize in remembering dimension. The other 32items (understanding, applying, and analysing) were developed based on situations included medication adherence, free treatment policy, routine examination in TB care, unified centralized management, return visit, standard treatment programs, commonly used drugs and side effects.

#### Phase 3: item selection

During the stage of item selection, we conducted three rounds of the Delphi survey to elicit expert opinion regarding the specific items that should be included in the CHLS-TB. The response rate was 100% in all rounds among 10 experts participating in this study. Almost 70% of them were male, and their age ranged from 39 to 68 years (Mean 47.63, SD 15.25). Their mean work experience in TB was 27.90 years (SD 14.26, ranging from 13 to 50 years).

In the first round, 71 items had a score of 4 or 5. Following the second round, 37 items were removed and 4 items were added. In the final round of rating, a total of 31 items were selected to form the CHLS-TB: 16 items in remembering section and 15 items in understanding, applying, and analyzing sections. In the remembering section, one point was given for each word that was read correctly, zero points were given for words that were read correctly or not read correctly. In the other sections, these selective items had one correct answer, a correct reply was worth three points, while an incorrect reply scored zero. Therefore, the maximum possible score for the scale was 61 points.

### Pilot study

In the pilot study, no major adjustments had to be made to the scale and all patients understood every item. Their feelings towards meaning of the scales accorded with the established purpose. It took about 10–15 min to complete the scale.

### Assessing the psychometric properties of the scale

The psychometric properties version of the CHLS-TB included a total of 31 items, which consist of 16 TB-related words in remembering section, and 15 items for understanding, applying, and analyzing sections. All psychometric tests were conducted using these items in the CHLS-TB.

#### Participants demographic characteristics

Participants were 511 individuals who complete the CHLS-TB, the response rate was 98.08% (511 out of 521), of which 498 were valid questionnaire. After 14 days, we received all information again (except for the demographic information) from 50 respondents. The respondents were aged 18–78 years and the mean age was 37.25 years (SD = 15.31). The other demographic details are shown in Table 1.

**Table 1:** Demographic characteristic of the participants (*n* = 498)

Characteristics	Total sample (*n* = 498)	Sample of EFA (n1 = 253)	Sample of CFA (n2 = 245)	*x*^*2*^/z	P
	n (%) or mean ± SD	n (%) or mean ± SD	n (%) or mean ± SD		
Age (range: 18–78 years)	37.25 ± 15.31	37.40 ± 15.45	37.09 ± 15.19	− 0.103	0.918
Gender				3.762	0.052
Male	300 (60.2%)	163(64.4)	137(55.9)		
Female	198 (39.8)	90(35.6)	108(44.1)		
Education				−1.827	0.068
Less than primary school	81 (16.3)	38(15.0)	43(17.6)		
Junior high school	122 (24.5)	55(21.7)	67(27.3)		
Senior high school	227 (45.6)	123(48.6)	104(42.5)		
College/university diploma or higher	68 (13.6)	37(14.6)	31(12.7)		
Marital status				2.543	0.468
Single	194 (39.0)	100(39.5)	94(38.4)		
Married	270 (54.2)	132(52.2)	138(56.3)		
Divorced	21 (4.2)	12(4.7)	9(3.7)		
Widowed	13 (2.6)	9(3.6)	4(1.6)		
Patient type				1.266	0.261
Outpatient	278 (55.8)	135(53.4)	143(58.4)		
Inpatient	220 (44.2)	118(46.6)	102(41.6)		
Medical history				1.404	0.495
Initial treatment	358 (71.9)	177(70.0)	181(73.9)		
Re-treatment	117 (23.5)	65(25.7)	52(21.2)		
Drug-resistant	23 (4.6)	11(4.3)	12(4.9)		
Treatment time				−0.671	0.502
1–2 months	306 (61.4)	152(60.1)	154(62.9)		
3–8 months	176 (35.3)	92(36.4)	84(34.4)		
> 8 months	16 (3.2)	9(3.6)	7(2.9)		
Occupation				12.010	0.100
Factory worker	71 (14.3)	34(13.4)	37(15.1)		
Agriculture	106 (21.3)	58(22.9)	48(19.6)		
Professional and technical personnel	48 (9.6)	30(11.9)	18(7.3)		
Office worker	11 (2.2)	5(2.0)	6(2.4)		
Business/service staff	49 (9.8)	17(6.7)	32(13.1)		
Student	74 (14.9)	44(17.4)	30(12.2)		
Retired	21 (4.2)	9(3.6)	12(4.9)		
Laid-off worker	48 (9.6)	20(7.9)	28(11.4)		
Others	70 (14.1)	36(14.2)	34(13.9)		
Census				2.591	0.274
Permanent residents	398 (79.9)	209(82.6)	189(77.1)		
Temporary residents	70 (14.1)	32(12.6)	38(15.5)		
Floating population	30 (6)	12(2.0)	18(7.3)		
Personal monthly income (RMB yuan)				−0.200	0.842
< 1999	192 (38.6)	95(37.5)	97(39.6)		
2000–2999	223 (44.8)	121(47.8)	102(41.6)		
3000–3999	70 (14.1)	32(12.6)	38(15.5)		
> 4000	13 (2.6)	5(2.0)	18(7.3)		

#### Content validity

In the final round of Delphi survey, the CVI ranged between 0.70 and 1.0 for each item, and the average of the CVI for all items on the scale was 0.95. The results meant that vast majority of experts evaluated the items on the scale with rating of “quite relevant” or “most relevant”, suggesting a strong agreement among the judges, and therefore high content validity.

#### Construct validity by EFA

**Fig. 1:**
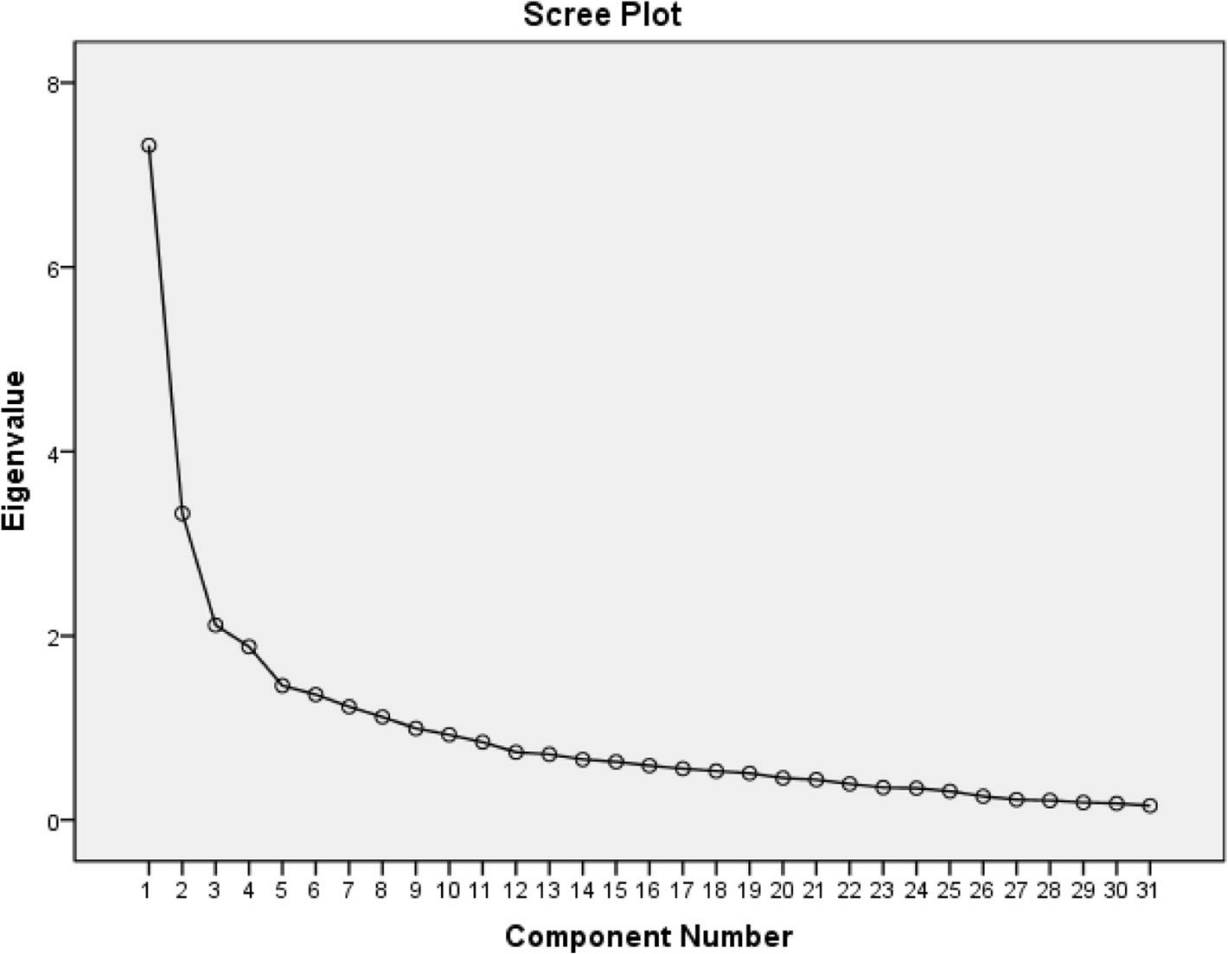
Scree plot of principal component factor analysis (*n* = 253) (Chinese Health Literacy Scale for Tuberculosis Patients)

The Kaiser-Meyer-Olkin (KMO) measure was 0.793, and Bartlett's test of sphericity was found to be significant (χ^2^ = 3295.802, *p* < 0.001), indicating the appropriateness of the data for EFA. The principal component factor analysis with varimax rotation and the scree plot yielded a four-factor solution (Table 2 and Fig. 1). B6 (What do you think is the key to cure tuberculosis?) was removed because its item-factor loading was < 0.40 (0.187 on factor 3 and 0.233 on factor 4). B15 (Which of the following conditions can get the qualified sputum specimens?) was removed because its item-factor loading was < 0.40 (0.145 on factor 3 and 0.327 on factor 4). Eventually, four factors with eigenvalues > 1 were generated and 29 items were retained. The eigenvalues ranged from 2.898 to 4.672. These four factors accounted for 47.254% of the total variance. All factor loading were > 0.40. As shown in Table 2, Factor 1 (10 items) and factor 2 (6 items), were consistent with read and memorize TB-related words, which were related to the remembering dimension that are envisaged according to the theoretical framework of the scale, and was retained as “Remembering 1(Words for TB-related drug names and main symptoms)” and “Remembering 2(Words for TB-related treatment and care adherence)”, respectively; Factor 3 (6 items), was associated with understand and explain the meaning of the health education leaflets or checklist, and examine if TB patients can apply the correct approach to the correct context, respectively, which was related to the understanding and applying dimension that are envisaged according to the theoretical framework of the scale, and was termed as “Understanding and Applying (Contexts for TB-related treatment and follow-up)”. Factor 4 (7 items) was related to the ability of TB patient to calculate and identify what unspecified assumptions are included in known conditions, which was related to the analyzing dimension that are envisaged according to the theoretical framework of the scale, and was termed as “Analysing (Ability for TB patients to calculate and analyze)”.

**Table 2:** Results of Exploratory Factor Analysis (*n* = 253)

Items		Factors			
		1	2	3	4
Factor 1 (F1): Remembering 1 (Words for TB-related drug names and main symptoms, 10 items)					
A11	Rifampicin	.742			
A3	Cough with blood	.727			
A2	Expectoration	.709			
A12	Bloody sputum	.673			
A4	Fever	.640			
A14	Respiratory infectious disease	.629			
A10	Isoniazid	.626			
A9	Tuberculosis	.549			
A16	Chemotherapy of tuberculosis	.513			
A1	Cough	.453			
Factor 2 (F2): Remembering 2 (Words for TB-related treatment and care adherence, 6 items)					
A8	Follow up		.755		
A7	Reexamination		.743		
A13	Sputum examination		.723		
A15	Close contact check		.676		
A6	Misses a dose		.568		
A5	Night sweats		.497		
Factor 3 (F3): Understanding and Applying (Contexts for TB-related treatment and follow-up, 6 items)					
B14	If you go for the follow-up on April 16, 2017, what time should you leave your night sputum?			.608	
B10	If there is a confirmed smear-positive pulmonary tuberculosis patients, what should he do with his own sputum?			.603	
B8	If a patient receiving an anti-tuberculosis chemotherapy has nausea and vomiting during the medication, but has not yet reviewed the time, what should the patient do?			.556	
B5	If you run out of drugs on April 12, 2017, what time should you go to the hospital to review and receive the medicine?			.541	
B7	If a patient who has received anti-tuberculosis chemotherapy for two months, his tuberculosis symptoms have basically disappeared, may he be able to reduce or disable the drug?			.539	
B9	Assume a smear-positive tuberculosis patients, his family appears the symptoms of coughing, sputum, etc., do you think his family should go to the hospital for further examination?			.463	
Factor 4 (F4): Analysing (Ability for TB patients to calculate and analyze, 7 items)					
B2	How many times should you take your medication every day?				.667
B4	How long this drug could be taken?				.643
B1	If you have a breakfast at 7:00 and should take the medication before a meal, what time do you have to take the medication?				.575
B11	Assume a patient who is in an infectious phase, could you tell me which of the following is wrong with him?				.570
B12	How many times do you need to check your sputum during the treatment of tuberculosis?				.564
B3	If your weight is 60 kg, how many tablets should you take each time?				.550
B13	If you are the first treatment of tuberculosis patients, what first time should you send sputum to review?				.447
Eigenvalues		4.672	3.894	3.185	2.898
% of variance		15.070	12.562	10.274	9.347
Cumulative%		15.070	27.632	37.906	47.254

#### Construct validity by CFA

**Fig. 2:**
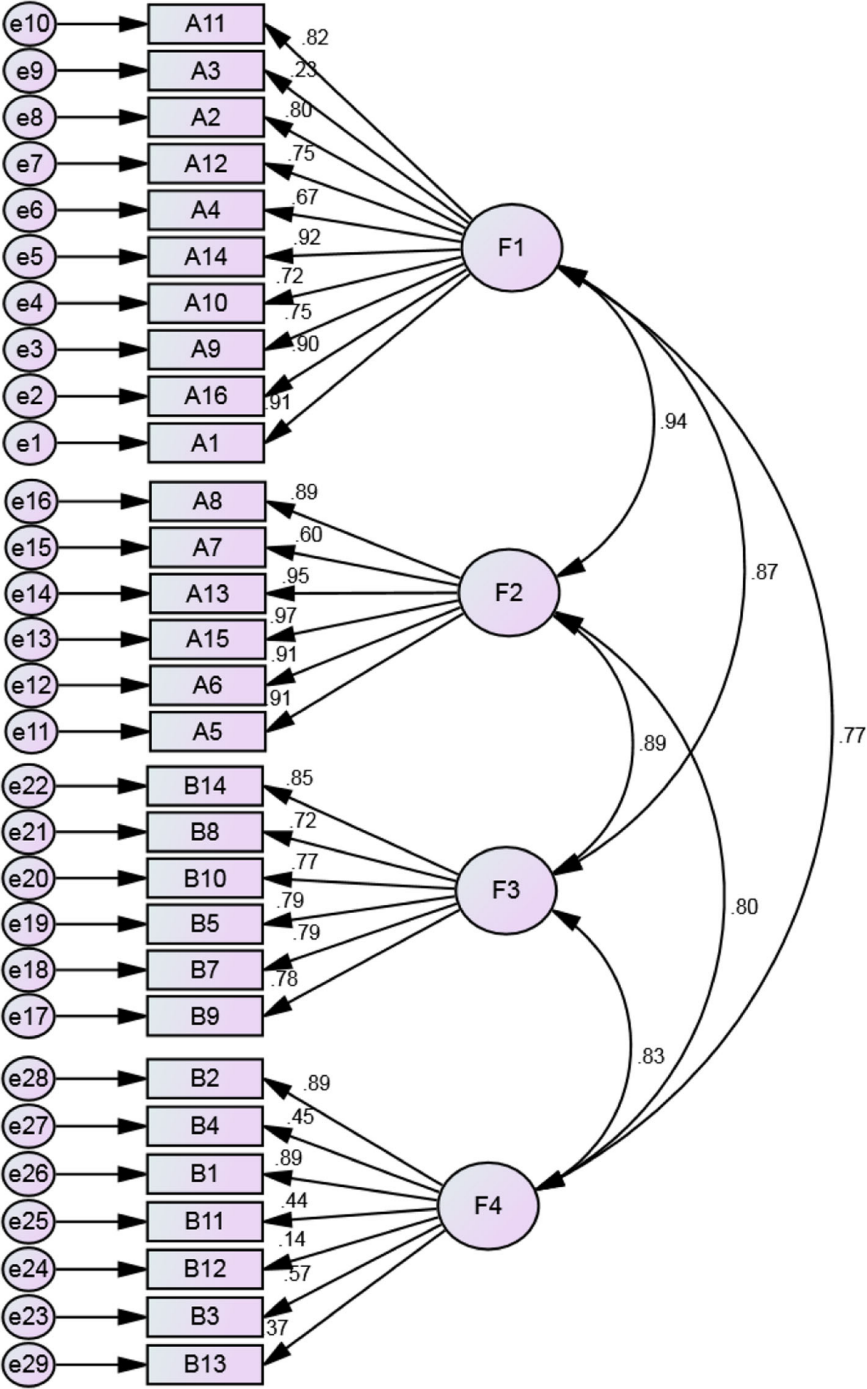
The standardized path diagrams of the confirmatory factor model (*n* = 245) (Chinese Health Literacy Scale for Tuberculosis Patients)

The confirmatory factor analysis revealed an acceptable fit of the four-factor model, with a GFI = 0.930, AGFI = 0.970, RMSEA = 0.069 (90% confidence interval 0.062–0.075), and χ2/df = 2.153 (χ2 = 759.867, df = 353). Figure 2 shows the standardized path diagrams of the confirmatory factor model.

#### Discriminant validity

The discriminant validity of the CHLS-TB is presented in Table 3, which shows the comparison of mean scores between the highest 27% (P_H_) and the lowest 27% (P_L_) scoring groups. The total mean(SD) score was 50.55(3.03) among those highest 27% (P_H_) scoring groups and 36.54(4.78)among those lowest 27% (P_L_) scoring groups. Each of the factors and total CHLS-TB had a statistically significant difference in the mean score value between the highest 27% (P_H_) and the lowest 27% (P_L_) of respondents (*p* < 0.01).

**Table 3:** Discriminant validity of the CHLS-TB

Variables	Range of scores	Mean ± SD	Mean ± SD		t
			Scores of P_H_	Scores of P_L_	
F1:Remembering 1	0–10	8.68 ± 2.18	9.77 ± 0.54	9.05 ± 1.39	6.84*
F2:Remembering 2	0–6	3.52 ± 2.23	5.45 ± 0.95	3.34 ± 2.05	13.20*
F3:Understanding and Applying	0–18	11.11 ± 4.97	15.95 ± 2.94	10.05 ± 3.92	16.56*
F4:Analysing	0–21	14.06 ± 5.85	19.39 ± 2.20	14.11 ± 4.35	15.33*
Total: CHLS-TB	0–55	37.37 ± 11.34	50.55 ± 3.03	36.54 ± 4.78	34.43*

*Statistically significant at p < 0.01

#### Reliability

The Cronbach’s alpha coefficient was 0.820 for the total scale and ranged between 0.722 and 0.857 for each factor (Table 4). The split-half reliability of CHLS-TB was 0.788. The total test-retest reliability coefficient over 2 week’s interval for the sub-sample of 50 participants was 0.958, and ranged between 0.843 and 0.968 for each factor (Table 4).

**Table 4:** Internal consistency and test-retest reliability of the CHLS-TB

Variables	No. of items	Cronbach’s α(*n* = 498)	Test-retest reliability (*n* = 50)
F1:Remembering 1	10	0.856	0.843*
F2:Remembering 2	6	0.857	0.899*
F3:Understanding and Applying	6	0.755	0.968*
F4:Analysing	7	0.722	0.955*
Total: CHLS-TB	29	0.820	0.958*

*Statistically significant at *p* < 0.01

#### Receiver operating characteristic (ROC) analysis

An ROC curve was used to analyze the classification of health literacy levels. Comparison of education levels indicated a cut-point of 45 for differentiating respondents with an education of at least college versus less than college level (sensitivity = 0.792, specificity = 0.721), and a cut-point of 35 for differentiating respondents with an education of at least junior high school versus less than junior high school level (sensitivity = 0.713, specificity = 0.620). Based on these analyses, we classified participants into three groups: adequate health literacy (score > 45), basic health literacy (score of 35–45), and low health literacy (score < 35). In our sample, 46.2% of participants were found to have low health literacy, 20.5% basic health literacy, and 33.3% adequate health literacy.

## Discussion

Tuberculosis is among the top 10 causes of death worldwide [8]. Low health literacy has been identified as one of the key elements of the social infrastructure of tuberculosis management and control [60]. Although numerous tools have been developed in an effort to measure health literacy in various contexts [61], research in CHLS-TB is scarce. The current study was to develop a theoretically driven instrument, the CHLS-TB, based on the revised Bloom’s taxonomy model and conducted its psychometric tests. The 133-item pool was generated initially using a literature review, focus group discussion, in-depth interview, and the pool subsequently reduced to 31 items after reviewed by experts.

The CHLS-TB encompasses a broad range of health literacy skills that TB patients need in order to treatment and management of TB infection (such as reading medication labels and warning, interpreting the test results, dosing of drugs, educational brochures) and is one of the few measures that evaluates health literacy through remembering comprehension. The skills included in the scale are all real-life situations that TB patients would encounter in clinical settings. The format of test items is diverse so as to allow the assessment of remembering, understanding, applying, and analysing skills. In addition, the scale was relatively easy to use, requiring only about 10–15 min to complete, and served as a quick assessment tool to identify TB patient’s individual health literacy levels as well as to provide pertinent information for clinicians to make patient-centered care plan.

### Psychometric properties of the CHLS-TB

Content validity, reflecting the adequacy of item sampling, refers to extent to which a specific set of items reflects a content domain [32]. The most commonly used and convincing approach for measuring content validity is the Delphi methods [62]. The findings of this study showed that the CVI values of each item ranged from 0.70 to 1, and the overall CVI value was 0.95, suggesting that CHLS-TB could clearly meet the requirement (CVI > 0.8) and authenticity regarding what it was supposed to measure [63].

EFA and CFA were undertaken to identify and confirm the presence of underlying construct. Exploratory factor analysis produced a clear four-factor solution, the item B6 (What do you think is the key to cure tuberculosis?) and B15 (Which of the following conditions can get the qualified sputum specimens?) was removed because its item-factor loading was < 0.40, the remaining 29 items had loading values > 0.40, suggesting that the underlying factors are meaningful. The confirmatory factory analysis results confirmed that a four-factor model was an acceptable fit to the data. Overall, the CHLH-TB corresponded very closely with what was predicted with the theoretical framework, and has a successful structure for measure TB patients’ health literacy at four different levels (remembering 1, remembering 2, understanding and applying, analyzing).

When the participants were categorized into the highest 27% (P_H_) group and the lowest 27% (P_L_) group based on the total scores, the discriminant validity was established. Our results showed that, the mean score of the lowest 27% (P_L_) group were significantly lower than those of the highest 27% (P_H_) group. These findings indicate that the CHLS-TB was able to uncover differences in health literacy between the highest 27% (P_H_) group and the lowest 27% (P_L_) group. The results suggest that the CHLS-TB, having good discriminative validity, is useful for comparing TB patients’ health literacy levels in cross-sectional studies. It can also assist clinicians in distinguishing between groups of TB patients with higher and lower health literacy.

The internal consistency of the CHLS-TB was acceptable (Cronbach’s α =0.820, split-half reliability = 0.788), and indicated a satisfactory degree of consistency between items and each dimension (Cronbach’s α =0.722 to 0.857). The Pearson correlation coefficient also indicated good consistency of the test results over time (r = 0.958, *p* < 0.01). Thus, this instrument has good reliability.ROC analysis indicated that the cut-off point for the instrument was set at 45 and 35. Thus, those with scores < 35 may require help in increasing TB health literacy, and those scores between 35 and 45 may require help in strengthening TB health literacy. The scale showed that nearly 50 % (46.2%) of the TB patients had low health literacy, which is a situation that cannot be ignored when attempting to control tuberculosis.

### Application value of the scale

The CHLS-TB is intended to be used for the following reasons. (a) This study reports on the development a new measure of health literacy based on the revised Bloom’s taxonomy model. We hope this will stimulate further debate about how the revised Bloom’s taxonomy model maybe translated into practical approaches to the further refinement of measurement of the health literacy. (b) The study highlights several potential challenges related to TB patient’s ability to understand daily TB-related treatment and self-management skills, such as reading labels, understanding the correct time and number of pills to take daily, remembering basic TB-related treatment terms used routinely in care in China. (c) This study builds on the limited literature to date about health literacy on tuberculosis. We found only three other studies focusing on measuring literacy on tuberculosis. One study investigated gender differences in health literacy about tuberculosis amongst South African high school students [64].Another study used health literacy approach including program planning and implementation with greater impact on TB control [65].The third study reported an urgent need to implement targeted interventions based on health literacy to educate TB patients for better TB control [66]. (d) This study provides a reliable and valid measurement to assess health literacy at both the individual and population levels.

### Limitation of the study

First, owing to the lack of established “gold criterion” for the measurement of tuberculosis specific health literacy, criterion validity could not be measured. Second, the TB patients were recruited from the same province, which might have no generalizability of the findings in this study. This study has been designed by a panel of 10 experts in developing the CHLS-TB, and a large sample of 498 participants to validate the scale, and the obtained scale with good psychometric properties. Therefore, it is a well-acceptable tool to measure health literacy on tuberculosis patients in China.

## Conclusion

In conclusion, 29-item of CHLS-TB with good reliability and validity were developed in this study. Different from the existing measurement tools, the CHLS-TB is the first to comprehensively consider the skills that TB patients need to master in various situations of treatment and life in China. The score of the scale ranges from 0 to 55. A score of 35 or less indicates that the patient is at a low level of health literacy; a score of 35 to 45 indicates that the patient has basic health literacy; a score of 45 or more indicates that the patient has a higher level of health literacy.

## Data Availability

The datasets used and/or analysed during the current study available from the corresponding author on reasonable request.

## Abbreviations

AGFI: Adjusted goodness of fit index
CFA: Confirmatory factor analysis
CHLS-TB: Chinese Health Literacy Scale for Tuberculosis Patients
CVI: Content validity index
DOTS: Directly observed treatment, short-course strategy
EFA: Exploratory factor analysis
FGD: Focus group discussion
GFI: Goodness-of-fit index
IOM: Institute of Medicine
KMO: Kaiser-Meyer-Olkin
NAAL: National Assessment of Adult Literacy
REALM: Rapid Estimate of Adult Literacy in Medicine
RMSEA: Root mean square error of approximation
ROC: Receiver operating characteristic analyses
SD: Standard deviation
TB: Tuberculosis
TOFHLA: Test of Functional Health Literacy in Adults (TOFHLA)
